# VEXAS Syndrome: Clinical Features, Hematologic Involvement, and Clinical Outcomes of Current and Emerging Therapies

**DOI:** 10.3390/hematolrep18030030

**Published:** 2026-04-23

**Authors:** Chanika Assavarittirong, Christopher Grant, Sandeep S. Nayak, Anthony L. Nguyen

**Affiliations:** 1 Department of Internal Medicine, Southwest Healthcare Medical Education Consortium, Temecula, CA 92590, USA; 2 Department of Hematology/Oncology, Moores Cancer Center, UC San Diego School of Medicine, UC San Diego Health, San Diego, CA 92037, USA; c1grant@health.ucsd.edu; 3 Department of Internal Medicine, Yale New Haven Health Bridgeport Hospital, Bridgeport, CT 06610, USA; dr.sandeepnayak@gmail.com; 4 Division of Hematology and Cellular Therapy, Moores Cancer Center, UC San Diego School of Medicine, UC San Diego Health, San Diego, CA 92037, USA; aln019@health.ucsd.edu

**Keywords:** VEXAS syndrome, UBA1 mutation, JAK inhibitors, myelodysplastic syndrome (MDS), allogeneic hematopoietic stem cell transplantation (allo-HSCT)

## Abstract

**Background/Objectives**: VEXAS (Vacuoles, E1-Enzyme, X-linked, Autoinflammatory, and Somatic) syndrome is a recently described adult-onset autoinflammatory disorder. It is characterized by somatic mutations in the UBA1 gene, systemic inflammation, macrocytic anemia, cytopenias, and bone marrow vacuolization and frequently overlaps with Sweet’s syndrome, relapsing polychondritis, and myelodysplastic syndrome (MDS). Because treatment options are evolving, we reviewed the current and latest evidence of clinical features and therapeutic methods. **Methods**: A comprehensive literature review was conducted using PubMed and MEDLINE for studies published between 1 January 2020 and 1 July 2025. Search terms included “VEXAS” and “treatment.” Eligible publications comprised clinical trials, multicenter and observational studies, and case reports containing therapeutic data. Findings were analyzed narratively with emphasis on treatment response, steroid-sparing effects, survival outcomes, and molecular responses. **Results**: Glucocorticoids remain the first-line therapy for acute management; however, this comes with near-universal steroid dependence. DMARDs and TNF-α inhibitors showed limited benefits. IL-6 inhibitors and JAK inhibitors showed improvement in overall response, with JAK inhibitors demonstrating a superior effect. Ruxolitinib showed a higher complete response rate and transfusion independence compared to other JAK inhibitors. Hypomethylating agents, particularly azacitidine, improved hematologic responses in patients with co-existing MDS and reduced UBA1 variant allele burden. Allogeneic hematopoietic stem cell transplantation may be the only current curative method, though with notable transplant-related mortality. **Conclusions**: JAK inhibitors and hypomethylating agents offer promising disease-modifying potential, while transplant may provide curative intent in selected patients. Ongoing clinical trials are taking place to dictate the treatment direction of VEXAS syndrome.

## 1. Introduction

**Figure 1:**
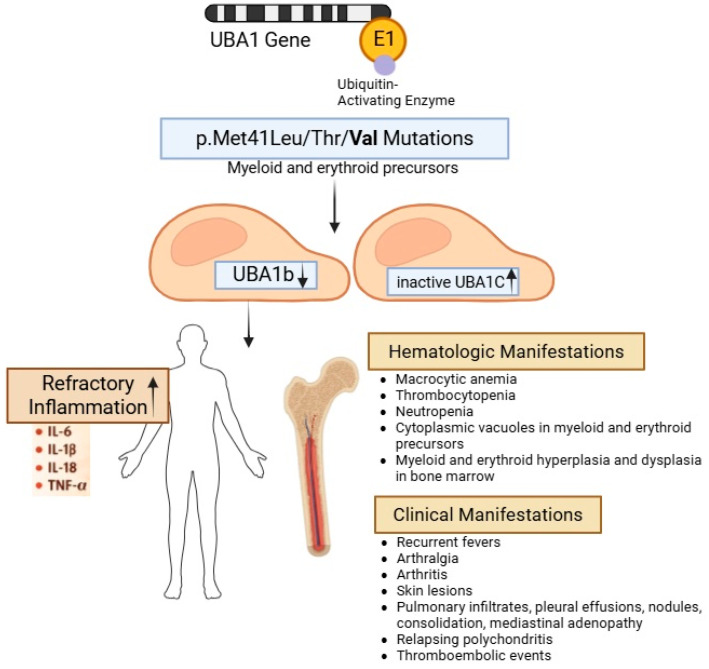
Pathophysiology and manifestations of VEXAS syndrome. Somatic mutations in the UBA1 gene at p/Met41 (Leu/Thr/Val) impair translation of the UBA1b cytoplasmic isoform, leading to expression of the inactive UBA1c isoform. The loss of UBA1b drives dysregulated ubiquitination and systemic inflammation, including the elevation of proinflammatory cytokines.

VEXAS (Vacuoles, E1-Enzyme, X-linked, Autoinflammatory, and Somatic) syndrome is a recently recognized, adult-onset, autoinflammatory disorder caused by somatic mutations in the UBA1 gene located on the X chromosome [1,2]. The UBA1 gene encodes the ubiquitin-activating enzyme E1, which catalyzes the first step in ubiquitination—an essential process in protein degradation and homeostasis. The syndrome was first described in 2020 by National Institutes of Health (NIH) researchers who reported 25 men with somatic mutations in UBA1 at methionine-41 (p.Met41) presenting with severe, treatment-refractory inflammatory syndromes. Variants affecting the start codon of UBA1 at p/Met41 (p.Met41Leu/Thr/Val) have been identified as pathogenic and are associated with a spectrum of refractory autoinflammatory syndromes. The p.Met41Val genotype leads to reduced translation of UBA1, especially the cytoplasmic isoform, UBA1b, in comparison to p.Met41Leu or Thr. The impaired translation of UBA1b results in the production of an alternative, inactive, UBA1c isoform, which becomes preferentially expressed when UBA1b synthesis is disrupted. The disease severity appears to be inversely correlated with UBA1b expression; however, current evidence suggests that the loss of UBA1b in the cytoplasmic space plays a more significant role in disease pathogenesis than the gain of the UBA1c isoform. The loss of ubiquitylated proteins leads to activation of inflammatory responses. Further studies show that the consequences of the mutated myeloid precursors lead to cytokine productions of IL-6, IL-1B, IL-18, and TNF-α, and tissue and systemic inflammation [3,4,5]. An illustration of the UBA1 mutations and their clinical and hematologic manifestations is summarized in Figure 1. Most reported patients are male with the onset at older than 50 years old. It is estimated that nearly one million individuals are affected worldwide, with 1 in 13,591 persons affected in North America, 1 in 4269 men older than 50 years, and 1 in 26,238 women older than 50 years [6,7]. These individuals exhibit systemic inflammation characterized by fevers, cytopenias, and cytoplasmic vacuoles in myeloid and erythroid precursors, often with dysplastic bone marrow findings.

Subsequent studies have expanded the clinical spectrum, showing involvement of the skin, lungs, cartilage, and vasculature, alongside systemic inflammatory features. In a Spanish cohort, macrocytic anemia, elevated ferritin, and elevated erythrocyte sedimentation rate (ESR) were the most prevalent findings [8]. Reported overlapping syndromes include relapsing polychondritis, Sweet’s syndrome, myelodysplastic syndrome (MDS), multiple myeloma, monoclonal gammopathy of undetermined significance, polyarteritis nodosa, and giant-cell arteritis [3]. Since its discovery, VEXAS syndrome has transformed understanding of adult-onset autoinflammation and hematologic overlap syndromes. This review synthesizes current evidence regarding clinical and hematologic features, treatment modalities, ongoing clinical trials, and future directions in the management of VEXAS syndrome.

### 1.1. Hematological Manifestation of VEXAS

The hematologic manifestations of VEXAS are hallmark features of the disease and a major diagnostic clue. Common findings include macrocytic anemia, thrombocytopenia, neutropenia, and cytoplasmic vacuoles in myeloid and erythroid precursors [3,9,10,11,12]. Macrocytosis is nearly universal, and bone marrow studies reveal vacuoles predominantly in early precursor cells—blasts, promyelocytes, and pronormoblasts—with fewer in monocytes, eosinophils, and plasma cells [10]. Bone marrow aspirates are typically hypercellular with myeloid and erythroid hyperplasia and varying degrees of dysplasia. Notably, vacuoles are often seen in smears but not in core biopsies.

Obiorah et al. reported macrocytic anemia in 100% of patients (16/16), absolute lymphopenia in 80%, monocytopenia and thrombocytopenia in 50%, and neutropenia in 13%, with myeloid malignancies present in 38% [10]. Associated hematologic disorders include MDS, plasma cell dyscrasias, macrophage activation syndrome/hemophagocytic lymphohistiocytosis, and monoclonal B-cell lymphocytosis. Up to 60% of patients harbor additional myeloid mutations, underscoring a strong link between UBA1 mutations and clonal hematopoiesis [10].

### 1.2. Clinical Manifestations of VEXAS

VEXAS predominantly affects men, given the X-linked nature of UBA1, but rare cases have been reported in women, including those with Turner syndrome [13,14]. Typical reported clinical manifestations include recurrent fevers in 65–100% of cases; arthralgia in up to 28%; arthritis in 58%; skin lesions in up to 84%; pulmonary infiltrates including ground glass opacities (87%), pleural effusion (53%), nodules (47%), consolidation (49%), and mediastinal adenopathy (58%); relapsing polychondritis in 52% of cases; and thromboembolic events [1,15]. Patients often meet diagnostic criteria for other rheumatologic disorders, delaying recognition. Cutaneous lesions are among the most common and may precede systemic findings [16]. Neurological manifestations such as encephalopathy, cerebral infarcts, posterior reversible encephalopathy syndrome, optic peri-neuritis, and tumefactive demyelination have been reported [17,18]. Though rare, cardiac manifestations have also been documented in a case series including pericarditis, myocarditis, heart failure with reduced ejection fraction, heart blocks, and amyloidosis [19]. The disease course is typically chronic and relapsing, with progressive cytopenias and inflammatory organ involvement contributing to morbidity. It is important to note that the first presentation of VEXAS syndrome may be difficult to differentiate from other rheumatological diseases.

## 2. Materials and Methods

A comprehensive literature review was conducted using PubMed and Medline, with searched terms “VEXAS” and “treatment” from 1 January 2020 to 1 July 2025. Eligible studies included clinical trials, clinical studies, multicenter studies, observational studies, randomized controlled trials, letters to editor, and case reports with therapeutic data. Data was synthesized narratively to emphasize therapeutic strategies, response rates, and evidence quality.

## 3. Results

**Figure 2:**
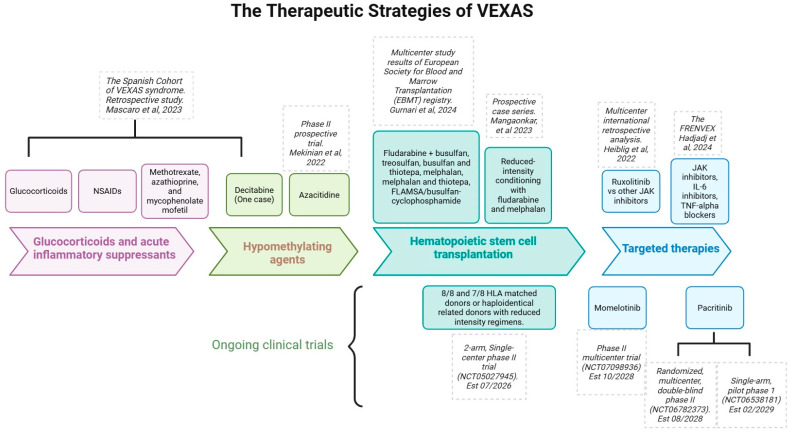
Comprehensive timeline of the therapeutic strategies of VEXAS, including glucocorticoids, hypomethylating agents, hematopoietic stem cell transplantation, targeted therapies, and ongoing clinical trials. Mascaro et al. 2023 [8]; Mekinian et al. 2022 [21]; Gurnari et al. 2024 [24]; Mangaonkar et al. 2023 [25]; Heiblig et al. 2022 [26]; Hadjadj et al. 2024 [27]; 2-arm, single-center phase II trial (NCT05027945) Est 07/2026 [28]; Phase III multicenter trial (NCT07098936) Est 10/2028 [29]; Randomized, multicenter, double-blind phase III (NCT06782373) Est 08/2028 [30]; Single-arm, pilot phase 1 (NCT06538181) Est 02/2029 [31].

The results of the comprehensive literature review which included glucocorticoids and acute inflammatory suppressants, targeted therapies with JAK and cytokine inhibitors, hypomethylating agents, and hematopoietic stem cell transplantation are summarized in Table 1. The timeline of the therapeutic strategies of VEXAS is picturized in Figure 2. Additional reviews of the ongoing clinical trials are summarized in Table 2.

**Table 1:** A summary of the clinical studies included in the treatment of VEXAS syndrome.

Type of Clinical Study	Number of Subjects	Implemented Methods	Clinical Outcomes	Reference
Retrospective cohort	N = 42 patients enrolled.	Genetic analyses, mosaicism distribution, and evaluation of presence of UBA1 mosaicism in non-hematopoietic tissue; outcome of medical treatments.	N = 30 included patients with UBA1 pathogenic variants. Glucocorticoids (prednisone 20 mg) most often administered. Only high doses alleviated the inflammatory symptoms, limited efficacy on hematologic responses. 70% complete response, partial 52.9%. One patient treated with decitabine 20 mg/m^2^ × 5 days; cycles of 28 days. Positive response after 4 cycles. 11th cycle with significantly reduced cystosolic vacuoles in promyelocytes and proerythroblasts in bone marrow aspiration. One patient underwent allogenic-HSC. Complete rescue in clinical phenotype and reversal of analytical abnormalities: 0% mutant allele fraction, ESR normalizing, hemoglobin and MCV normalized. Anti-IL-6 and JAK inhibitors showed complete responses of 12.5% and 20%, respectively. Anti-TNF with 100% negative response rate. Anti IL-1 with partial response of 60%, anti-CD20 with 75% partial response. Colchicine, methotrexate, mycophenolate, azathioprine, cyclophosphamide, and IVIGs with more than 50% negative response rate.	Mascaro et al. [8]
Multicenter, cross-sectional, retrospective study	N = 39	Spanish cohort study with genetic analysis, treatment and outcomes.	Glucocorticoids were used in all patients and response rate was improved. IL-6 and JAK inhibitors showed the highest response rates (75% and 76.92%, respectively).	Garcia-Escudero et al. [20]
Phase II prospective trial	N = 29	Azacitidine in steroid-dependent VEXAS syndrome associated with myelodysplastic syndrome (MDS) and chronic myelomonocytic leukemia (CMML).	Patients with IPSS-R int 2/high or IPSS-R low with significant cytopenia received azacitidine 75 mg/m^2^ for 7 days every 4 weeks for at least 6 cycles. 17/29 patients with hematologic response (8 complete responses, 9 stable diseases). UBA1-mutated patients with quick response in inflammatory-related symptoms.	Mekinian et al. [21]
Retrospective cohort	N = 4	5-Azacytidine in VEXAS syndrome to de-escalate corticosteroids and UBA1 mutation burden.	Azacytidine treatment resulted in reduction in UBA1 clonal burden and reduced steroid dependence in all 4 patients. All 4 patients showed resolution of inflammatory symptoms on no steroids or low dose within 1–3 cycles. Two were able to stop the steroids completely and 2 remained on 2 mg and 5 mg daily prednisolone. In one patient, no UBA1 was detected at 24 months. All 4 patients had MDS with low blast type.	Trikha et al. [22]
Retrospective cohort	N = 4	Allogeneic hematopoietic stem cell transplant in the UK.	2 patients with MDS-associated VEXAS and 2 without MDS. All presented with macrocytic anemia. All 4 were treated with corticosteroids. One patient developed septic shock and cardiac arrest and passed away 11 days post-transplantation. One patient achieved disease control but developed post-transplant myelitis resulting in paraplegia, and passed away 11 months post-transplantation. One patient developed complications post-transplantation with hemophagocytic lymphohistiocytosis, aseptic encephalitis, and EBV reactivation. One patient remained alive and well, in remission 40 months post-transplant.	Al-Hakim et al. [23]
Retrospective cohort	N = 19	Allogeneic hematopoietic cell transplantation.	68% with concomitant MDS, 63% matched unrelated donor, 16% matched related donor, 5% mismatched unrelated donor, 16% mismatched related donor. Allo-HCT performed at a median of 41 months from VEXAS onset diagnosis. Reduced-intensity regimen was used in 14 patients (74%). Median follow-up of 14 months from allo-HCT, 2-year overall survival was 74.2%, transplant-related mortality of 25.8%. No patient had VEXAS or MDS/MPN relapse (N = 11).	Gurnari et al. [24]
Prospective case series	N = 5	Allogeneic hematopoietic stem cell transplantation using uniform approach and graft versus host disease prophylaxis strategy.	Consistent plan of reduced-intensity fludarabine and melphalan conditioning in all 5 patients. Donors were either matched sibling donors or matched unrelated donors. None had recurrent inflammatory symptoms or worsening cytopenias post-transplantation. All patients were alive and at the time of publication, none developed grade II-IV acute GVHD or chronic GVHD.	Mangaonkar et al. [25]
Clinical trial, retrospective multicenter study	N = 30	Ruxolitinib vs. other JAK inhibitors (tofacitinib, baricitinib, and upadacitinib).	Ruxolitinib median follow-up of 6.9 months, 75% (9/12) still receiving treatment vs. 28% (5/18) for other JAK inhibitors. Median time to the next line of treatment was not reached in ruxolitinib; 3.3 months (95% CI, *p* < 0.001) in patients treated with other JAKis. Higher response rates with ruxolitinib at 1 month (CR 67% vs. 38%, *p* = 0.13), 3 months (CR 83% vs. 18%, *p* = 0.001) and 6 months (CR 87% vs. 11%, *p* = 0.002). Mean hemoglobin at 3 months > 0.9 g/L. All 4 treated with ruxolitinib who were previously dependent on RBC transfusion no longer required transfusion. 2 patients treated with ruxolitinib had MDS progression. At last follow-up, 3 patients were off steroids in ruxolitinib group and one in other JAKi group (upadacitinib).	Heiblig et al. [26]
Retrospective study	N = 110	Efficacy and safety of targeted therapy in VEXAS from FRENVEX.	At 3 months, highest overall response with IL-6 inhibitors (32%), followed by JAK inhibitors. 0% overall response with TNF-alpha inhibitors. At 6 months, the highest overall response was seen in JAK inhibitors with 30%, and 26% in IL-6 inhibitors. Survival was statistically longer at 24 months with JAK inhibitors, followed by IL-6 inhibitors (*p* < 0.0001).	Hadjadj et al. [27]

**Table 2:** A summary of four ongoing clinical trials for VEXAS syndrome which include studies of pacritinib, momelotinib, and allogeneic hematopoietic stem cell transplant.

Type of Study	Therapeutic Option	Methods	Estimated Time of Completion	Trial
Single-arm pilot, phase 1 [31]	Pacritinib	15 patients to be enrolled in 28-day cycle of pacritinib up to 200 mg twice daily. Primary endpoint: dose-limiting toxicities.	February 2029	NCT06538181
Randomized, double-blind, multicenter, phase II (PAXIS) [30]	Pacritinib	Pacritinib dose A vs. dose B vs. placebo. Primary outcome: overall clinical response.	August 2028	NCT06782373
Phase II multicenter [29]	Momelotinib	57 patients with or without MDS. Exploring clinical response (complete and partial responses).	October 2028	NCT07098936
Single-center phase II [28]	Allogeneic hematopoietic stem cell transplant	VEXAS syndrome refractory to treatment. 8/8 or 7/8 HLA-matched related or unrelated donors or a haploidentical related donor. Reduced-intensity regimens.	July 2026	NCT05027945

### 3.1. Glucocorticoids and Acute Inflammatory Suppressants

Glucocorticoids remain the cornerstone and the first line of acute management. In a Spanish cohort of 30 patients, glucocorticoids achieved complete or partial response in all cases (70% complete), while NSAIDs and methotrexate produced partial responses in fewer than one-third. Azathioprine and mycophenolate mofetil had limited efficacy [8]. Although glucocorticoids are effective in rapidly suppressing inflammation, nearly all patients develop steroid dependence or relapse upon tapering.

### 3.2. Targeted Therapies: JAK and Cytokine Inhibitors

The FRENVEX multicenter retrospective study (N = 110) evaluated the comparative efficacy of targeted agents [24]. At three months, IL-6 inhibitors had the highest response rate (32%), followed by JAK inhibitors (24%); by six months, JAK inhibitors achieved superior response rates (30%) and longer survival at 24 months (*p* < 0.0001).

Ruxolitinib, a selective JAK1/JAK2 inhibitor, demonstrated superior outcomes compared with other JAK inhibitors (tofacitinib, baricitinib, and upadacitinib) in a multicenter study of 30 patients [21]. At six months, 87% of patients receiving ruxolitinib achieved a complete response, compared with 11% in the comparator group (*p* = 0.002). Ruxolitinib also enabled transfusion independence in all previously dependent patients and reduced corticosteroid requirements. Its efficacy may relate to dual JAK1/2 inhibition and favorable pharmacodynamics compared to JAK1/3-targeting agents. Nonetheless, MDS progression was observed in a minority, underscoring the need for long-term surveillance [26,32,33].

### 3.3. Hypomethylating Agents

#### 3.3.1. Azacitidine

Trikha et al. [22] reported four patients with VEXAS-associated MDS who achieved marked reduction in UBA1 variant allele burden and resolution of inflammation within one to three cycles of azacitidine; two discontinued steroids completely. A French registry cohort of 11 patients treated with azacitidine showed a 46% clinical response and significant reductions in C-reactive protein and steroid use [34]. In a prospective phase II trial of 29 patients with steroid-dependent VEXAS and MDS/CMML, azacitidine achieved hematologic responses in 59% [21]. These data support azacitidine as an effective steroid-sparing agent and potential disease-modifying therapy.

#### 3.3.2. Decitabine

One study described a Spanish cohort of VEXAS syndrome in various therapeutic options. An isolated case of one patient who was treated with decitabine showed significant improvement after 4 cycles and reduced vacuolization by the 11th cycle, with improvement in hemoglobin level, MCV and ESR normalizing, and a significant reduction in mutant allele fraction percentage. This study was described in the above section with glucocorticoids and DMARDs [8].

### 3.4. Hematopoietic Stem Cell Transplantation

Allogeneic hematopoietic stem cell transplantation (allo-HSCT) is currently the only curative strategy. In a multicenter cohort of 19 patients (68% with MDS), allo-HSCT achieved 74.2% two-year overall survival, with 25.8% transplant-related mortality [24]. Complete molecular remission was observed within six months, and no relapses occurred. A UK cohort of four patients reported variable outcomes: one long-term remission and three deaths or severe complications [23].

A prospective case series using standardized reduced-intensity conditioning with fludarabine and melphalan achieved 100% survival and no grade II–IV graft-versus-host disease [25]. These results emphasize the potential curative role of allo-HSCT but highlight the need for optimized conditioning regimens and patient selection.

### 3.5. Current Ongoing Clinical Trials

#### 3.5.1. JAK Inhibitors

##### Pacritinib

A single-arm, pilot phase 1 study is evaluating the safety and tolerability of pacritinib in patients with VEXAS syndrome (NCT06538181). A total of 15 patients are estimated to be enrolled in a dose expansion to the target goal of pacritinib 200 mg twice daily for a continuous 28-day cycle. The primary endpoint will be dose-limiting toxicities [31]. This study’s secondary outcomes will include changes in WBC, ANC, hemoglobin, and platelets; hematologic improvements using IWG 2018 criteria; and existence of the UBA1 variant allele. This study has been actively recruiting since February 2025 and is estimated for completion in February 2029.

Currently, there is another ongoing randomized, multicenter, double-blind phase II study (NCT06782373) evaluating patients with inflammatory VEXAS syndrome who have required ongoing glucocorticoid therapy for 4 consecutive weeks, requiring between 15 and 45 mg daily of prednisone equivalence. A total of 78 patients is the enrollment goal. Individuals will be stratified by prescribed glucocorticoid dose and randomized to receive pacritinib dose A, pacritinib dose B, or placebo. The specified targeted doses for dose A and dose B have not been reported. Overall clinical response defined as a symptomatic clinical response after 24 weeks is the primary outcome. Secondary outcomes are measured proportions of patients on each arm achieving clinical biochemical response, partial clinical response, and stable disease after 24 weeks. Crossover after week 24 will be allowed for individuals assigned to placebo. The study is estimated to be completed in August 2028 [30].

##### Momelotinib

A phase II multicenter trial (NCT07098936) is assessing momelotinib in approximately 57 patients with or without MDS. After 24 weeks of momelotinib initiation, clinical response will be determined via complete (disappearance of symptoms related to systemic inflammation and daily dose of steroids < 10 mg/d of equivalent prednisone) and partial responses (complete disappearance of symptoms of systemic inflammation according to treating physician and reduction of at least 50% of daily dose steroids compared to baseline or daily dose > 10 mg/d prednisone equivalent). Other means of additional secondary outcomes will include steroid dose reduction, overall survival, MDS evolution/progression, duration, best clinical response, RBC transfusion independence, and UBA1 evolution variant. This study is estimated to be completed in October 2028 [29].

#### 3.5.2. Allogeneic Hematopoietic Stem Cell Transplant

A single-center phase II trial (NCT05027945) is being conducted for patients with VEXAS syndrome refractory to treatment who have met the criteria for transfusion dependence for anemia or thrombocytopenia, have platelets < 75,000 or neutropenia < 1000/μL, or have a WHO-defined myeloid neoplasm. Individuals must also have an 8/8 or 7/8 HLA-matched related or unrelated donor or a haploidentical related donor [28]. The study aims to explore two arms: 8/8 and 7/8 HLA-matched donors, or haploidentical related donors, with reduced-intensity regimens for each. Individuals will receive fludarabine and busulfan if they are 8/8 HLA-matched donors for conditioning and will receive fludarabine, cyclophosphamide, and busulfan if they are 7/8 HLA-matched donors or haploidentical donors. The post-transplant GVHD prophylaxis will also be standardized for both arms. Cyclophosphamide will be administered at 50 mg/kg IV once daily on days +3 and +4, followed by mycophenolate mofetil from day +5 through day +45, and tacrolimus from day +5 until approximately day +180. Primary outcomes will be measured with reversal of clinical VEXAS phenotype at 1 and 2 years post-HSCT, and sustained donor engraftment at 100 days and 1 year post-HSCT. Secondary outcomes will include the safety profile, focusing on transplant-related toxicities, as well as the incidence of grade III–IV acute GVHD and moderate-to-severe chronic GVHD [28]. The study is ongoing and is estimated to be completed in July 2026. The ongoing clinical trials are summarized in Table 2.

#### 3.5.3. Treatment Algorithm

**Figure 3:**
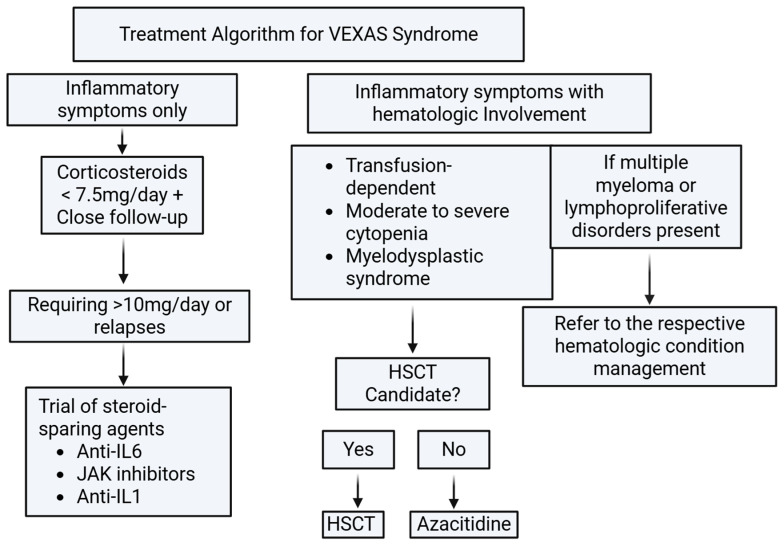
Treatment algorithm summary based on ACR Consensus 2025.

The American College of Rheumatology (ACR) proposed a consensus-based treatment algorithm for VEXAS syndrome in 2025. In patients with isolated inflammatory manifestations, initiation of corticosteroids with close follow-up is recommended. The key determinant guiding treatment escalation is steroid requirement. Patients requiring more than 10 mg/day of corticosteroids or experiencing recurrent relapses should be considered for steroid-sparing therapies, including anti–IL-6, JAK inhibitors, or anti–IL-1 agents. In cases where inflammatory symptoms coexist with hematologic manifestations—such as transfusion dependence, moderate-to-severe cytopenias, or myelodysplastic syndrome (MDS)—evaluation for hematopoietic stem cell transplantation (HSCT) candidacy is recommended. For patients who are not candidates for HSCT, treatment with azacitidine may be considered. Additionally, if hematologic involvement includes multiple myeloma or other lymphoproliferative disorders, management should follow disease-specific hematologic treatment guidelines [35]. The 2025 ACR consensus-based treatment algorithm is summarized in Figure 3.

## 4. Discussion

VEXAS syndrome represents a paradigm shift in adult-onset autoinflammatory diseases, linking somatic hematopoietic mutations with systemic inflammation. However, current therapeutic strategies remain largely empirical, based on retrospective cohorts and case reports. Glucocorticoids are effective for acute control but unsustainable long term due to dependency, toxicity, and failure to address the underlying clonal pathology. Conventional DMARDs and biologics (TNF-α, and IL-1 blockade) offer inconsistent and limited benefits, while IL-6 inhibitors and JAK inhibitors demonstrate more durable responses in subsets of patients. A recent aggregated review of JAK inhibitor use across cohorts reported roughly one-third complete responses and an additional ~30% partial responses, reinforcing class efficacy but also underscoring heterogeneous benefit and the need to identify predictors of response [36].

Among JAK inhibitors, ruxolitinib shows the most robust evidence for efficacy and steroid-sparing potential [26,27]. However, variability in response and persistence of hematologic disease highlights the complex interplay between clonal hematopoiesis and inflammation. This emphasizes that targeting the inflammation alone may be insufficient in many patients. Hypomethylating agents, particularly azacitidine, show promise in patients with concomitant MDS or refractory inflammation, with molecular responses suggesting modification of the underlying clonal process [19,26,27]. More recent series further support azacitidine’s dual benefit—mitigating inflammatory activity while improving cytopenia—with response rates exceeding 60% in some real-world cohorts; these data strengthen a pragmatic approach of reserving HMAs for patients with MDS features or steroid-refractory disease [27,37]. In parallel, the FRENVEX registry continues to show that JAK inhibitors and IL-6 blockade outperform other targeted agents at medium-term follow-up, yet durable steroid-free remission remains uncommon. It remains unclear whether these responses translate into durable disease modification or merely clonal suppression [27].

Allogeneic transplantation is currently the only potential curative therapy, achieving eradication of UBA1 clones and durable remission in selected patients [24,25]. Nonetheless, transplant-related mortality remains significant, particularly in older patient populations with comorbidities, underscoring the need for standardized reduced-intensity regimens and improved GVHD prophylaxis. The ongoing phase II trial [28] may define the feasibility of this approach for broader clinical application. Emerging multicenter data from EBMT and allied groups suggest that standardized reduced-intensity conditioning can deliver acceptable two-year survival and high rates of full donor chimerism, though acute GVHD and infectious complications remain key determinants of outcome; these signals support earlier referral for transplant evaluation in biologically high-risk patients [24,38].

### 4.1. Future Directions

Two prospective JAK2-focused programs are particularly relevant. First, pacritinib—uniquely inhibiting both JAK2 and IRAK1 (a proximal mediator of innate immune signaling)—is in active investigation (phase I pilot and a randomized phase II with steroid-sparing endpoints), a mechanistically attractive strategy given the autoinflammatory biology of VEXAS [31]. Second, momelotinib (with activity against JAK1/2 and ACVR1) is being studied in a multicenter phase II trial; early registry experience hints at concurrent improvement in anemia and inflammatory markers, which, if confirmed, could be advantageous in cytopenic phenotypes [29]. Collectively, these trials should clarify whether JAK2-dominant inhibition (±IRAK1) confers superior steroid-free remission, transfusion independence, and reductions in UBA1 variant allele fraction (VAF) compared with broader JAK blockade.

### 4.2. Safety Priorities

Serious infections have been cataloged in national VEXAS registries, particularly under JAK inhibition and in patients with high inflammatory burden, so future studies should embed standardized infection prophylaxis, viral reactivation monitoring, and dose-modulation algorithms, which are currently inconsistently applied across studies [39].

### 4.3. Trial Design and Endpoints

A major limitation across the existing literature is the lack of standardized endpoints. Future trials should consider incorporating outcomes such as (i) durable steroid-free clinical remission, (ii) hematologic improvement (e.g., transfusion independence per IWG 2018 criteria), (iii) biomarker response (CRP/ESR and ferritin trajectories), and (iv) molecular response (decline in UBA1 VAF), to link symptom control with clonal modification. Stratification by disease biology, “inflammation-predominant” versus “MDS-predominant”, and by co-mutational background may also sharpen signal detection and guide sequencing (e.g., JAK inhibitor first for inflammatory dominance and HMA first for MDS-dominant disease, with crossover for non-responders) [38,40].

### 4.4. Transplant Positioning

Given converging evidence that allo-HSCT can eradicate UBA1-mutant clones, consensus frameworks are needed to define transplant candidacy and timing (e.g., age/fitness, transfusion dependence, marrow blast percentage, and inflammatory control at conditioning) and to standardize reduced-intensity backbones with GVHD prophylaxis tailored for older adults [24,41].

### 4.5. Mechanistic Opportunities

Finally, mechanistic work linking aberrant translation of cytoplasmic UBA1 isoforms to innate immune activation supports evaluation of upstream pathway inhibitors (e.g., IRAK1/MyD88 axis) and combination strategies (JAK2 ± IRAK1 plus HMA) in biomarker-enriched cohorts; this is hypothesis-generating at present, but testable within adaptive trial designs.

## 5. Conclusions

Therapeutic management of VEXAS syndrome remains challenging. In clinical practice, the management algorithm emphasizes early diagnosis and confirmation of UBA1 mutations, initial control with glucocorticoids, rapid transition to targeted therapy in relapsing disease or when coexisting hematologic disorders are present, and timely consideration of HCST. Looking forward, the integration of biomarker-driven stratification, including inflammatory markers and UBA1 variant allele burden, will be critical for guiding treatment decisions and monitoring disease trajectory. Glucocorticoids provide reliable short-term control but are not a sustainable strategy due to steroid dependence and cumulative toxicity. Conventional immunosuppressive agents offer limited benefits. Targeted therapies—particularly JAK inhibitors and IL-6 blockade—have demonstrated more meaningful and durable responses, with ruxolitinib emerging as a leading steroid-sparing option in inflammatory-predominant disease. In patients with concomitant MDS or refractory cytopenias, hypomethylating agents such as azacitidine show dual hematologic and inflammatory benefit.

Allogeneic hematopoietic stem cell transplantation remains the only potentially curative approach, capable of eradicating UBA1-mutant clones and inducing durable remission. However, transplant-related morbidity and mortality necessitate careful patient selection and optimization of conditioning strategies.

Ongoing clinical trials investigating JAK2-focused therapies and standardized transplant protocols will be critical for establishing evidence-based treatment algorithms. Future studies should prioritize biomarker-driven stratification, harmonized response criteria, and earlier identification of high-risk patients.

## Data Availability

No new data were created or analyzed in this study.

## Abbreviations

The following abbreviations are used in this manuscript:

VEXAS	Vacuoles, E1-enzyme, X-linked, autoinflammatory, and somatic
MDS	Myelodysplastic syndrome
Allo-HSCT	Allogeneic hematopoietic stem cell transplantation
GVHD	Grafts-versus-host disease
